# Losing a parent to cancer as a teenager: Family cohesion in childhood, teenage, and young adulthood as perceived by bereaved and non‐bereaved youths

**DOI:** 10.1002/pon.5163

**Published:** 2019-07-18

**Authors:** Dröfn Birgisdóttir, Tove Bylund Grenklo, Tommy Nyberg, Ulrika Kreicbergs, Gunnar Steineck, Carl J. Fürst

**Affiliations:** ^1^ Faculty of Medicine, Department of Clinical Sciences Lund, Oncology and Pathology, Institute for Palliative Care Lunds University Lund Sweden; ^2^ Clinical Cancer Epidemiology, Department of Oncology‐Pathology Karolinska Institute Stockholm Sweden; ^3^ Department of Caring Science University of Gävle Gävle Sweden; ^4^ Centre for Cancer Genetic Epidemiology, Department of Public Health and Primary Care University of Cambridge Cambridge UK; ^5^ Department of Women's and Children's Health Karolinska Institutet Stockholm Sweden; ^6^ Department of Caring Sciences, Palliative Research Center Ersta Sköndal Bräcke University College Stockholm Sweden; ^7^ The Sahlgrenska Academy, Department of Oncology, Division of Clinical Cancer Epidemiology University of Gothenburg Institute of Clinical Sciences Gothenburg Sweden

**Keywords:** adolescents, bereavement, cancer, family cohesion, oncology, parental death, teenagers, young adults

## Abstract

**Objective:**

The aim of this study was to investigate levels of perceived family cohesion during childhood, teenage years, and young adulthood in cancer‐bereaved youths compared with non‐bereaved peers.

**Methods:**

In this nationwide, population‐based study, 622 (73%) young adults (aged 18‐26) who had lost a parent to cancer 6 to 9 years previously, when they were teenagers (aged 13–16), and 330 (78%) non‐bereaved peers from a matched random sample answered a study‐specific questionnaire. Associations were assessed using multivariable logistic regression.

**Results:**

Compared with non‐bereaved youths, the cancer‐bereaved participants were more likely to report poor family cohesion during teenage years (odds ratio [OR] 1.6, 95% CI, 1.0‐2.4, and 2.3, 95% CI, 1.5‐3.5, for paternally and maternally bereaved youths, respectively). This was also seen in young adulthood among maternally bereaved participants (OR 2.5; 95% CI, 1.6‐4.1), while there was no difference between paternally bereaved and non‐bereaved youths. After controlling for a number of covariates (eg, year of birth, number of siblings, and depression), the adjusted ORs for poor family cohesion remained statistically significant. In a further analysis stratified for gender, this difference in perceived poor family cohesion was only noted in females.

**Conclusion:**

Teenage loss of a parent to cancer was associated with perceived poor family cohesion during teenage years. This was also noted in young adulthood among the maternally bereaved. Females were more likely to report poor family cohesion. Our results indicate a need for increased awareness of family cohesion in bereaved‐to‐be families with teenage offspring, with special attention to gender roles.

## 1 INTRODUCTION

Losing a parent is one of the most tragic experiences that can occur in the life of a child or adolescent.^1^ Bereaved children and youths have been shown to be at higher risk of negative consequences, such as anxiety, depression,^2^ self‐injury,^3^, ^4^ premature death,^5^ and suicide attempts^6^ compared with their non‐bereaved peers.

In the literature, the most constant factors that can counteract the negative impact of bereavement are warmth and connection between the surviving parent and the bereaved child, the mental health of the surviving parent, and family functioning.^7^, ^8^ One of the core elements of family function is family cohesion, which is a broad concept intended to grasp the sense of emotional bonding between family members but also includes other factors, such as support and feeling of togetherness.^9^ Poor family cohesion has been shown to be associated with anxiety and depression^10^ and to predict higher stress responses in adolescent children of cancer‐patients.^11^ It has similarly been associated with increased mental health problems in parentally bereaved children.^12^, ^13^ Furthermore, family cohesion mediates the effects of parental bereavement on adolescents.^7^, ^14^ Also, in previous reports from this project, poor family cohesion has been strongly associated with adverse outcome.^15^

Family cohesion changes with time and is affected by situational stressors and changes in developmental needs as the children matures.^9^ To be able to support bereaved children and adolescents in an efficient way, there is a need for more knowledge about which contextual family‐ and health care‐related factors impact their well‐being.^16^ Only limited evidence exists on the impact bereavement has on the family as a unit and its function.^17^ Further, there is a dearth of knowledge on the relationship between bereavement and family cohesion, as perceived by youths themselves.

The aim of this study was to investigate the levels of perceived family cohesion during childhood, teenage years, and young adulthood in youths who had lost a parent to cancer in their teenage years, 6 to 9 years prior to the study, compared with their non‐bereaved peers.

## 2 METHODS

For inclusion in this nationwide, population‐based study, the bereaved participants needed to have lost a parent from cancer during their teenage years (at 13‐16 years of age). The participants were identified through the Multi‐Generation Register at Statistics Sweden by using information about the lost parents from the Swedish National Cause of Death Register. For inclusion, the lost parents had to have died before the age of 65 in the years 2000 to 2003 and been diagnosed with cancer at least 2 weeks before the death. The participant had to have been registered at the same address as both parents, and the other parent needed to be alive at the time of follow‐up.

A random sample of non‐bereaved participants was identified by Statistic Sweden at a ratio of 1:2 (non‐bereaved:cancer‐bereaved). Participants in the non‐bereaved group were matched by age, gender, and place of residency**.** All parents were non‐divorced. All participants needed to be born in one of the Nordic countries, to understand Swedish, to have an identifiable telephone number, and live in Sweden at the time of the study.

### 2.1 Data collection

Data collection started with an invitation letter to all participants who met the inclusion criteria, followed by an information call from a research assistant. If oral consent was given, the anonymous questionnaire, an ethics information sheet, and a reply card was sent. Information about participants' right to withdraw from the study at any time was given both orally and in writing. All participants gave oral and written consent. The reply card was returned separately in order to keep the questionnaires anonymous. Afterwards, a thank you/reminder card was sent followed by reminder phone calls to those who had not returned their reply card.

### 2.2 Measurements

The data was collected through a study‐specific questionnaire that was developed according to well‐established routines.^18^, ^19^ This included developing the items in the questionnaire based on the literature, expert recommendations, previous questionnaires from the research group, and foremost on the topics brought up in semi‐structured interviews with bereaved youths (n = 16). The single‐item questions and response alternatives were tested for face validity with 15 cancer‐bereaved and two non‐bereaved young adults. The concept of “family cohesion,” which in the Swedish language is straightforward, was well understood. None of the participants made any remarks regarding this question throughout the process. The feasibility of the study was then tested in a pilot study. The questionnaire included a total of 271 items, of which 21 were considered relevant for this study (n = 5 family cohesion, n = 16 potential confounding variables).

The perception of family cohesion was evaluated with five single items, with the question:

*Did you as a family have good cohesion* during:

- your childhood (until you were approximately 11‐12 years old)?
- your teenage years (until the death of your parent)?
- 0‐6 months after your loss?
- 7‐12 months after your loss?
- today?

There were four response alternatives: “No, not at all” and “Yes, little” (labelled poor family cohesion), “Yes, moderate” and “Yes, very good” (labelled good family cohesion).

The question for the non‐bereaved participants, for whom there was no loss, had only one time‐frame for the teenage years. To enable comparison of the perceived family cohesion during teenage years, between the groups, the non‐bereaved participants got subquestions (b), (c), and (d) combined into one variable. Reporting poor family cohesion at one or more of these three teenage time‐frames in question, was labelled as poor family cohesion during teenage years for the cancer‐bereaved participants.

### 2.3 Data analysis

The IBM SPSS Statistics 23.0 software (IBM Corp., Armonk, N.Y., USA) was used for statistical analyses. Crude odds ratios (ORs) with 95% CIs were calculated with bivariable logistic regression. To control for possible confounding factors, 16 possible confounding variables that were considered relevant to family cohesion or bereavement were preselected on the basis of literature review and previous analysis within the research project.^4^, ^15^ A forward selection (likelihood ratio test) was performed on the preselected variables. All variables that met the entry criterion of *P* < .25 at one or more of the time periods under investigation were then included in a multivariable logistic regression model used to calculate adjusted ORs with 95% CIs (Table A1). The adjusted ORs and 95% CIs for each time period were calculated with the model in three steps, every step adding more variables to the model. Further, analysis was made on the data stratified by the matching variables and also by gender of the deceased parent. Our comparisons were two‐tailed and performed at the.05 significance level, apart from the forward selection (likelihood ratio test) which had the entry criterion at.25 significance level.

### 2.4 Ethical considerations

The study was approved by the Regional Ethical Review Board of Karolinska Institute, Stockholm, Sweden (2007/836‐31). To minimize the risk of causing distress to the participants, the data was not collected during holidays or during the anniversary month of participants' parental loss. The overwhelming majority of the participants perceived their participation in the study as meaningful and positive.^20^

## 3 RESULTS

A total of 1272 young adults (18‐26 years old) met the criteria for inclusion and were asked to participate in the study. Of these, 622 (73%) cancer‐bereaved individuals, 337 of whom had lost their father and 284 their mother, and 330 (78%) non‐bereaved individuals returned the questionnaire. Participants' characteristics are displayed in Table 1.

**Table 1:** Characteristics of the participants

	Cancer‐Bereaved^b^		Non‐Bereaved^c^
	n (%)		n (%)
Confirmed eligible^a^	851		421
Not reachable	55 (6.5)		24 (5.7)
Declined participation	66 (7.8)		28 (6.6)
Did not return the questionnaire	108 (12.7)		39 (9.3)
Participated (response rate)	622 (73.1)		330 (78.4)
	Paternally bereaved	Maternally bereaved	
Gender of the deceased parent			
Male (father)	337 (54.3)		–
Female (mother)		284 (45.7)	–
Not stated^d^	1		–
Gender			
Male	170 (50.4)	139 (48.9)	169 (51.2)
Female	167 (49.6)	145 (51.1)	161 (48.8)
Year of birth			
1988‐1990	123 (36.7)	87 (30.6)	119 (36.2)
1986‐1987	149 (44.5)	137 (48.2)	146 (44.4)
1984‐1985	63 (18.8)	60 (21.1)	64 (19.4)
Not stated^d^	2		1
Birth order			
Firstborn	75 (22.3)	69 (24.3)	104 (31.7)
Middle	88 (26.2)	60 (21.1)	87 (26.5)
Youngest	155 (46.1)	146 (51.4)	127 (38.7)
No siblings	18 (5.4)	9 (3.2)	10 (3.1)
Not stated^d^	1		2
Current employment status^e^			
Studying at high school level	16/332 (4.8)	8/281 (2.8)	13/325 (4.0)
Adult education at high school level	19/332 (5.7)	12/280 (4.3)	18/325 (5.5)
Studying at university level	88/332 (26.5)	99/280 (35.4)	112/327 (34.2)
Employed or self‐employed	199/335 (59.4)	155/280 (55.4)	182/326 (55.8)
Unemployed	44/334 (13.2)	47/281 (16.7)	53/323 (16.4)
On parental leave	3/332 (0.9)	6/280 (2.1)	2/324 (0.6)
On sick leave	3/332 (0.9)	4/280 (1.4)	4/324 (1.2)
Residential region			
Rural	23 (6.9)	31 (11.0)	30 (9.1)
Small village or town	72 (21.6)	41 (14.5)	60 (18.3)
Mid‐sized town	146 (43.7)	137 (48.6)	156 (47.6)
City of more than 500 000	93 (27.8)	73 (25.9)	82 (25.0)
Not stated^d^	3	2	2
Father's year of birth			
1960‐	27 (8.3)	33 (12.0)	63 (19.4)
1955‐1959	11 (34.3)	93 (34.8)	111 (34.3)
1950‐1954	109 (33.6)	75 (28.1)	93 (28.7)
‐1949	32 (9.9)	46 (17.2)	57 (17.6)
Not stated^d^	12	9	6
Mother's year of birth			
1960‐	72 (22.2)	53 (19.9)	112 (35.2)
1955‐1959	111 (34.3)	93 (34.8)	118 (37.1)
1950‐1954	109 (33.6)	75 (28.1)	64 (20.1)
‐1949	32 (9.9)	46 (17.2)	24 (7.6)
Not stated^d^	13	17	12

a All those identified by the registers who met the inclusion criteria.

b Young adults who lost a parent to cancer between the ages of 13 and 16 years in Sweden, 2000‐2003.

c A random sample from the Swedish population, matched for age, sex, and residency to the cancer‐bereaved young adults.

d The group “not stated” is not included in calculations of prevalence.

e Participants were allowed to report more than one alternative.

**Figure 1:**
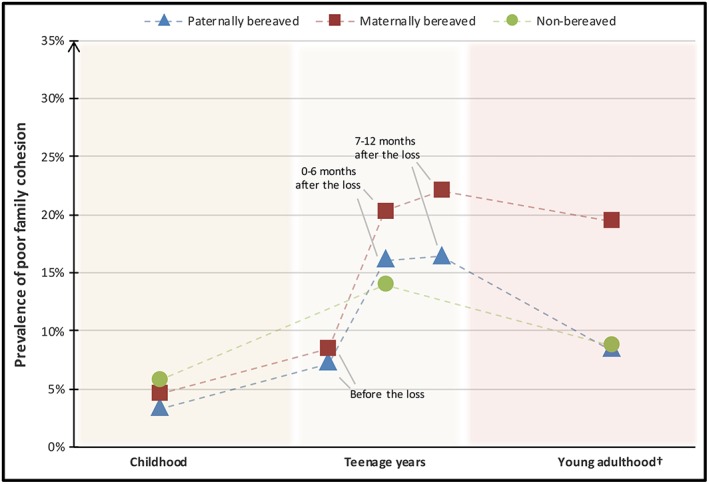
Prevalence of perceived poor (no/little) family cohesion among non‐bereaved and cancer‐bereaved youths at different time periods^**. †**^At the time of the survey (aged 18–26). Note. For graphical reasons, only the frequencies between 0% and 35% are included in the Figure.

The responses of the vast majority of both cancer‐bereaved and non‐bereaved participants indicated good (moderate to very good) family cohesion during childhood, while 3% to 6% of the participants self‐assessed the family cohesion in this period as poor (no or little) (Figure 1). Higher prevalence of perceived poor family cohesion was reported in all groups during the teenage years. In total, 20.3% of the paternally bereaved and 27.3% of the maternally bereaved participants reported poor family cohesion at one or more of the time periods during the teenage years, while 14.0% of the non‐bereaved reported poor family cohesion during the teenage years. When asked about family cohesion today, ie, at the time of the survey in young adulthood, 8.4% of the paternally bereaved participants reported poor family cohesion, while the prevalence was at 19.5% among those who had lost their mother, in comparison with 8.8% of the non‐bereaved youths (Figure 1).

Table 2 shows crude ORs as well as adjusted ORs, with corresponding 95% CIs, for the reported poor family cohesion during childhood, teenage years and young adulthood. There was no statistically significant difference in reported perception of family cohesion between the groups during childhood. However, during the teenage years, the cancer‐bereaved youths were more likely to report poor family cohesion compared with their non‐bereaved peers: for the paternally bereaved youths, the crude OR was 1.6 (95% CI, 1.0‐2.4) and for maternally bereaved youths, 2.3 (95% CI, 1.5‐3.5). In young adulthood (6‐9 years after the loss), the difference in perceived poor family cohesion was statistically significant for those who had lost their mother, with OR 2.5 (95% CI, 1.6‐4.1), in comparison with the non‐bereaved participants. The difference was not statistically significant for the paternally bereaved participants for this time period.

**Table 2:** Perceived family cohesion in cancer‐bereaved and non‐bereaved youths at different time periods

	Family Cohesion			Unadjusted	Adjusted^a^		
	Poor (no or little) n/total (%)	Good (moderate or good) n/total (%)	Missing n (%)	OR (95% CI)	OR_Adjustment1_ (95% CI)	OR_Adjustment2_ (95% CI)	OR_Adjustment3_ (95% CI)
Childhood							
Cancer‐bereaved	24/619 (3.9)	595/619 (96.1)	3 (0.5)	0.7 (0.4‐1.2)	0.7 (0.4‐1.2)	0.6 (0.3‐1.1)	0.7 (0.3‐1.3)
–Paternally bereaved	11/336 (3.3)	325/336 (96.7)	1 (0.3)	0.6 (0.3‐1.2)	0.6 (0.3‐1.2)	0.5 (0.2‐1.1)	0.6 (0.2‐1.2)
–Maternally bereaved	13/282 (4.6)	269/282 (95.4)	2 (0.7)	0.8 (0.4‐1.6)	0.8 (0.4‐1.6)	0.7 (0.3‐1.5)	0.8 (0.4‐1.7)
Non‐bereaved	19/329 (5.8)	310/329 (94.2)	1 (0.3)	1.0 [reference]	1.0 [reference]	1.0 [reference]	1.0 [reference]
Teenage years							
Cancer‐bereaved	145/618 (23.5)	473/618 (76.5)	4 (0.6)	**1.9 (1.3‐2.7)**	**1.9 (1.3‐2.7)**	**1.8 (1.2‐2.7)**	**2.0 (1.3‐3.0)**
–Paternally bereaved	68/335 (20.3)	267/335 (79.7)	2 (0.6)	**1.6 (1.0‐2.4)**	**1.6 (1.0‐2.4)**	**1.5 (1.0‐2.4)**	**1.7 (1.1‐2.7)**
–Maternally bereaved	77/282 (27.3)	205/282 (72.7)	2 (0.7)	**2.3 (1.5‐3.5)**	**2.2 (1.5‐3.4)**	**2.2 (1.4‐3.4)**	**2.4 (1.5‐3.8)**
Non‐bereaved	46/329 (14.0)	283/329 (86.0)	1 (0.3)	1.0 [reference]	1.0 [reference]	1.0 [reference]	1.0 [reference]
Young adulthood^b^							
Cancer‐bereaved	82/611 (13.42)	529/611 (86.6)	11 (1.8)	**1.6 (1.0‐2.5)**	**1.6 (1.0‐2.5)**	**1.6 (1.0‐2.6)**	**1.6 (1.0‐2.6)**
–Paternally bereaved	28/333 (8.4)	305/333 (91.6)	4 (1.2)	1.0 (0.6‐1.6)	1.0 (0.6‐1.7)	1.0 (0.6‐1.7)	1.0 (0.5‐1.7)
–Maternally bereaved	54/277 (19.5)	223/277 (80.5)	7 (2.5)	**2.5 (1.6‐4.1)**	**2.4 (1.5‐4.0)**	**2.5 (1.5‐4.1)**	**2.5 (1.5‐4.2)**
Non‐bereaved	29/330 (8.8)	301/330 (91.2)	0 (0.0)	1.0 [reference]	1.0 [reference]	1.0 [reference]	1.0 [reference]

Abbreviations: CI, confidence interval; OR, odds ratio.

a Variables added to the logistic regression model and used to calculate the adjusted ORs: OR_Adjustment 1_: gender, year of birth, residential region. OR_Adjustment 2_: variables from OR_Adjustment 1_ + birth order, number of siblings, mother's year of birth, father's year of birth, educational level of mother, educational level of father, ever been bereaved of a sibling, depression in at least one parent, alcohol/drug misuse in at least one parent. OR_Adjustment 3_: variables from OR_Adjustments 1 & 2_ + have been bullied, have been physically assaulted or sexually violated, have ever been diagnosed with depression.

b Young adulthood: At the time of the survey (aged 18–26).

After the step‐wise adjustments for the teenage time period, all adjusted ORs for poor family cohesion remained statistically significantly higher for the bereaved compared with the non‐bereaved group, and varied between 1.5 (95% CI, 1.0‐2.4) and 1.7 (95% CI, 1.1‐2.7) among the paternally bereaved and between 2.2 (95% CI, 1.5‐3.4) and 2.3 (95% CI, 1.5‐3.8) among the maternally bereaved youths. In young adulthood, the reported perception of poor family cohesion among those who had lost a mother was statistically significantly higher compared with that among the non‐bereaved participants, resulting in an adjusted OR of 2.3 (95% CI, 1.3‐3.9) after the final adjustments (Table 2).

The analysis stratified by age or place of residency showed no substantial changes to the main results (data not shown). However, the cancer‐bereaved females had a significantly higher risk of reporting poor family cohesion during teenage years, compared with the non‐bereaved females, (OR: paternally bereaved: 2.7 [95% CI, 1.3‐3.8], maternally bereaved: 3.2 [95% CI, 1.8‐5.5]); and in young adulthood for the maternally bereaved females (OR: 3.5 [95% CI, 1.8‐7.1]). No statistically significant difference was found between the cancer‐bereaved and non‐bereaved male participants.

## 4 DISCUSSION

In this nationwide, population‐based study, we found an association between the loss of a parent to cancer and poor family cohesion during the teenage years. Moreover, those who had lost their mother were more likely to report poor family cohesion also in young adulthood, 6 to 9 years after the loss. These results remained statistically significant even after adjustments for several possible confounding factors. A gender specific analyses showed that these results were statistically significant only for the female participants.

To the best of our knowledge, no previous studies have reported on family cohesion changes over time, as perceived by parentally‐bereaved offspring. Factors involved in family cohesion, such as communication, emotional connection, perceived support, and relationships within the family, might possibly explain the increased prevalence of poor family cohesion among the bereaved participants.

Previous research has showed that family function is based on the interaction between individuals in the family, and when one dies, the others need to adapt to a new constellation,^21^ affecting the whole family system. The relationship dynamics between the surviving parent and child change after the death of a parent.^22^ This is supported in a long‐term follow‐up study, which showed that parentally bereaved youths had less harmonious relations with their surviving mother or father, including lack of communication, compared with their peers in non‐bereaved families.^23^ The relationship with the surviving parent has been shown to be a major factor influencing the children's coping skills and well‐being.^7^, ^22^, ^24^ The surviving parent is him or herself going through bereavement and emotional difficulties that may affect the capability of giving emotional support to their children or conducting positive parenting.^24^, ^25^

Our results also show that among the maternally bereaved youths, the perception of poor family cohesion appeared to continue into young adulthood, years after the loss of the mother. Among the paternally bereaved participants, however, the level of perceived family cohesion in young adulthood did not differ from that in the non‐bereaved controls. Studies have shown that widowed fathers have more difficulties in adapting to life after a partner's death,^26^ while women have better coping strategies when adjusting to bereavement.^27^ Communication, emotional bonding, and support are some of the core components of family cohesion,^9^ and in comparison with mothers, widowed fathers have been shown to be less likely to communicate about emotions,^25^, ^28^ provide positive parenting,^28^ or react to the children's loss‐related needs.^25^ However, Werner‐Lin and Biank argue that the difference seen in the family adaptation to loss of a parent may be based, not on the gender of the surviving parent, but, rather, on the role the surviving parent played in the family's life preceding the illness and the death.^29^

Further analysis on the basis of the gender of the participant showed higher levels of perceived poor family cohesion among the bereaved female participants compared with the non‐bereaved females, while no significant difference was found between the male participants. Family relationships have been shown to be especially prominent to female adolescents' well‐being,^30^ and they experience more emotional distress as a reaction to poor family cohesion compared with boys.^31^ Bereaved girls have also been shown to have greater likelihood to internalize problems^1^ and greater vulnerability than boys^1^, ^14^ as well as a stronger likelihood to take on more responsibility for the family life.^14^ Our results indicate that an awareness may be needed for bereaved‐to‐be families with teenagers according to their role in the family and gender.

The large sample size and high participation rate (73%‐78%) are the main strengths of this nationwide, population‐based study. Another strength was the well‐prepared and comprehensive questionnaire that was based on qualitative interviews with both bereaved and non‐bereaved young adults.

Throughout the study process, an epidemiological framework adapted to this field of research was followed.^32^ To enable adjustments, we assessed numerous possible confounding factors. When examining possible confounding factors during the data analysis phase, we performed an initial sorting by examining them one by one in relation to the outcome with a generous cut‐off level (0.25) to maximize the possibility of finding factors that would explain our findings.

The questionnaire was designed using one direct question per phenomenon, where all questions were directly related to the real‐life phenomena under investigation. This, enabled a comprehensive collection of data on teenagers' experience when losing a parent to cancer.

The comprehensive concept of family cohesion was self‐assessed through a subjective global measurement. All of the existing validated instruments for family cohesion included a large number of items and none of them was validated for our target group at the time of data collection. In line with that, a recent systematic review of self‐report family assessment measures stated that all of the validated instruments use a large number of items and no evidence exists of their responsiveness to changes in family functioning over time.^33^

Using a global‐single‐item question can sometimes be more preferable when measuring a complex phenomenon than using answers from a multiple‐item scale that have been computed into one single rating.^34^ This allows the participants to weigh into their own assessment those aspects of the phenomenon that are relevant to them.^34^

Since the comprehensive concept of family cohesion was self‐assessed through a subjective global measurement, we cannot exactly define what family cohesion means for each participant. However, we assume that at the moment of answering the questionnaire, the feeling is real to the participant. Furthermore, none of the participants, made any remarks regarding the concept of family cohesion during the face‐validity interviews. They all seemed to have a clear picture of what family cohesion meant to them.

### 4.1 Study limitations

Our study design implies the possibility of recall‐induced bias regarding data from the childhood and teenage time periods. On the other hand, to collect the data prospectively was not considered as an option because of practical, economical, and ethical reasons. Furthermore, a recent study investigating the accuracy of retrospective reports on family environment as experienced by adolescence found that retrospective and prospective reports agreed well regarding the emotional dimensions of the family life (such as family cohesion), that can be well captured with retrospective reports.^35^

We also have no knowledge about whether the level of family cohesion differed between our participants and the young adults who declined participation in our study, and the generalizability of our findings may not be applicable outside our setting and population.

### 4.2 Clinical implications

Our findings showed that losing a parent to cancer as a teenager increases the risk of poor family cohesion as perceived by parentally bereaved youth. Impaired family cohesion has been shown to be associated with a number of negative outcomes for adolescents.^15^, ^36^ Hopefully, our findings will encourage clinicians caring for dying parents with teenage offspring to pay attention to the family cohesion, to identify those at increased risk of poor family cohesion in bereavement, and to provide support as needed. According to the results of two systematic reviews, supportive interventions can benefit bereaved‐to‐be families with minor children, although further research is still needed.^37^, ^38^ It has been shown that an intervention such as “The Family Bereavement Program” can strengthen the relationship between the surviving parent and the bereaved child or adolescent, which can have a positive effect on both the parent's and the child's health and well‐being.^24^, ^39^, ^40^

## 5 CONCLUSIONS

In this nationwide, population‐based study, we found that for teenagers, losing a parent to cancer increases the risk of poor family cohesion during the teenage years, when compared with non‐bereaved peers. The perception of poor family cohesion lasted into young adulthood among the maternally bereaved youths. However, these findings were only noted among females. These results warrant further investigations of family cohesion among youths facing bereavement, including influencing factors within the family, as well as bereavement support.

## FUNDING SOURCE

The Swedish Cancer Foundation [2008–758]; the Gålö Foundation; the Kamprad Family Foundation for Entrepreneurship; and the Mats Paulssons Stiftelse supported the research project.

## CONFLICT OF INTEREST STATEMENT

The authors have no conflict of interest to report.

## DATA AVAILABILITY STATEMENT

The data that support the findings of this study are not publicly available because of legal and ethical restrictions as described by the Swedish law and ethical boards regarding data of sensitive nature. This is in order to assure data confidentiality and to protect the privacy of the research participants.

## APPENDIX A

### Overview of the preselected variables and variables associated with reported family cohesion at different time periods

**Table:**

Childhood	Forward Selection P a	Teenage Years	Forward Selection P a	Young adulthood^b^	Forward Selection P a
Background variables of the participant, added at step one (adjustment 1)					
Gender		✓Gender	.001	Gender	
Year of birth		✓Year of birth	.102	Year of birth	
Residential region		✓Residential region	.151	✓Residential region	.040
Religious or spiritual		Religious or spiritual		Religious or spiritual	
Background variables of parents and family‐related variables, added at step two (Adjustment 2)					
Number of siblings		✓Number of siblings	.006	Number of siblings	
✓Birth order	.250	✓Birth order	.211	Birth order	
✓Mother's year of birth	.128	Mother's year of birth		✓Mother's year of birth	.020
Father's year of birth		Father's year of birth		✓Father's year of birth	.001
Educational level of mother		✓Educational level of mother	.090	Educational level of mother	
Educational level of father		✓Educational level of father	.180	Educational level of father	
Ever been bereaved of a sibling		✓Ever been bereaved of a sibling	.140	Ever been bereaved of a sibling	
Depression in at least one parent		✓Depression in at least one parent	.005	Depression in at least one parent	
✓Alcohol/drug misuse in at least one parent	<.001	✓Alcohol/drug misuse in at least one parent	<.001	✓Alcohol/drug misuse in at least one parent	.003
Adverse events added at step three (Adjustment 3)					
✓Have experienced being bullied	.025	✓Have experienced being bullied	.002	✓Have experienced being bullied	.001
✓Have experienced being physically assaulted or sexually violated	.008	✓Have experienced being physically assaulted or sexually violated	<.001	✓Have experienced being physically assaulted or sexually violated	.129
Have ever been diagnosed with depression		✓Have ever been diagnosed with depression	.250	✓Have ever been diagnosed with depression	.035

a The P values are based on forward selection (likelihood ratio test) with the entry criterion of P < .25.

b At the time of the survey (today), when participants were aged 18 to 26 years.

✓ Variables included in the multivariable logistic regression model after meeting the entry criterion of the forward selection (likelihood ratio test).
